# Lemniscal Corticothalamic Feedback in Auditory Scene Analysis

**DOI:** 10.3389/fnins.2021.723893

**Published:** 2021-08-19

**Authors:** Natsumi Y. Homma, Victoria M. Bajo

**Affiliations:** ^1^Center for Integrative Neuroscience, University of California, San Francisco, San Francisco, CA, United States; ^2^Coleman Memorial Laboratory, Department of Otolaryngology – Head and Neck Surgery, University of California, San Francisco, San Francisco, CA, United States; ^3^Department of Physiology, Anatomy and Genetics, University of Oxford, Oxford, United Kingdom

**Keywords:** descending projections, layer VI cortical neurons, thalamus, medial geniculate body, tonotopy, harmonicity, speech, music

## Abstract

Sound information is transmitted from the ear to central auditory stations of the brain via several nuclei. In addition to these ascending pathways there exist descending projections that can influence the information processing at each of these nuclei. A major descending pathway in the auditory system is the feedback projection from layer VI of the primary auditory cortex (A1) to the ventral division of medial geniculate body (MGBv) in the thalamus. The corticothalamic axons have small glutamatergic terminals that can modulate thalamic processing and thalamocortical information transmission. Corticothalamic neurons also provide input to GABAergic neurons of the thalamic reticular nucleus (TRN) that receives collaterals from the ascending thalamic axons. The balance of corticothalamic and TRN inputs has been shown to refine frequency tuning, firing patterns, and gating of MGBv neurons. Therefore, the thalamus is not merely a relay stage in the chain of auditory nuclei but does participate in complex aspects of sound processing that include top-down modulations. In this review, we aim (i) to examine how lemniscal corticothalamic feedback modulates responses in MGBv neurons, and (ii) to explore how the feedback contributes to auditory scene analysis, particularly on frequency and harmonic perception. Finally, we will discuss potential implications of the role of corticothalamic feedback in music and speech perception, where precise spectral and temporal processing is essential.

## Introduction

In everyday life we are constantly analyzing our acoustic environment, which is filled with sounds from many different sources. For example, we can listen to a person next to us while others in the room are chatting. With little effort, we can treat the voice of that talker as the desired foreground signal and segregate it from the background of all other sounds in the room. We can also listen to the melody in a symphony while focusing on the parts played by different musical instruments. These listening abilities are based on the process of “auditory scene analysis” (Bregman, 1990), which is essential for grouping or segregating sound mixtures into perceptually meaningful categories while perceiving the whole auditory environment.

In auditory scene analysis, we categorize different, simultaneously occurring sounds based on several acoustic properties such as onset asynchrony (Darwin, 1984; Darwin and Ciocca, 1992), harmonicity (Moore et al., 1985; Hartmann et al., 1990), and spatial information (Cherry, 1953; Eramudugolla et al., 2008). We can also group sound sequences, for example, based on their rhythmic structure (Miller and Heise, 1950; Bregman and Campbell, 1971; van Noorden, 1975). In addition to exploiting various acoustic attributes, scene analysis relies on attention, learning and memory and incorporates information from other sensory systems (Shamma et al., 2011; Maddox et al., 2015; Atilgan et al., 2018).

**FIGURE 1:**
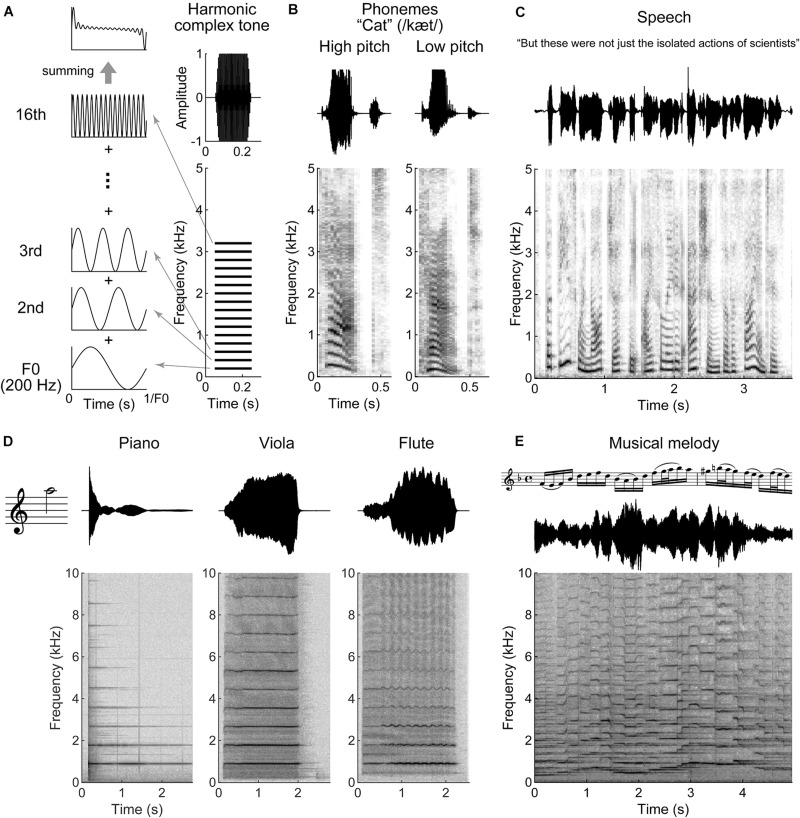
Harmonic structures in speech and music. **(A)** Schematic illustration of a harmonic complex tone comprised 200 Hz fundamental frequency (F0) with 16 harmonics (right bottom). The 16 harmonics are integer multiples of F0. The right top indicates a total amplitude waveform of the tone. The left panels show waveforms of F0, 2nd, 3rd, and 16th harmonics. The periodicity of the harmonic complex tone, the sum of all the harmonics (left top panel), is equal to F0. **(B–E)** Waveforms (top) and spectrograms (bottom) for a segment of speech and music. **(B)** Female (high pitch) and male (low pitch) voices pronouncing, “cat.” The harmonic structures are observed around the vowel “a” (æ). **(C)** A segment of speech. Syllables occur every ∼0.2 to 0.5 s (2–5 Hz). **(D)** The instrumental tones played by piano, viola, and flute at 880 Hz F0 (A5 note). Although the tones evoke the same pitch sensation, their timbre differs as indicated with the waveforms and spectrograms. **(E)** A segment of musical melody played by violin. The tone pitch fluctuates faster than speech in this example (it could be slower dependent on tempo and rhythm).

Human speech, animal vocalizations, and the sounds produced by many musical instruments are all periodic sounds comprised of a fundamental frequency (F0) plus its multiple integer harmonics (Figure 1). This condition is referred as “harmonicity” and is one of the basic acoustic properties for auditory scene analysis. In addition, harmonicity is considered as a basis of the perceptual attribute, “pitch,” that allow us to order sounds from low to high. Intonation, rising or falling of pitch, expresses grammatical meaning or emotion in speech. Pitch also helps in discriminating voices of different speakers, identifying different musical instruments, or conveying the melodic line in music. F0 differences between concurrent vowels contribute to indicate and segregate different talkers (Culling and Darwin, 1993; de Cheveigné, 1995; Arehart et al., 2011). The harmonic components of vowels show a unique energy distribution of frequencies, known as the spectral envelope. The peaks in the envelope (“formant frequencies”) characterize phonemes and provide critical spectral information to discriminate them (Klatt, 1982; Swanepoel et al., 2012). Similarly, the spectral envelope is characteristic for an individual musical instrument and defines its sound quality (“timbre”) (Figure 1D; Town and Bizley, 2013). Harmonicity and spectral regularity can serve as a strong grouping cue and play a key role for music and speech perception (McDermott and Oxenham, 2008; Micheyl and Oxenham, 2010). The harmonic structure of the sound spectrum helps the listener keep track of a speaker in competing, simultaneous speech signals (de Cheveigné et al., 1997; Popham et al., 2018). Moreover, harmonicity can contribute to longer lasting memory storage by connecting several aspects of spectral information to the behavior of a single attribute, namely F0 (McPherson and McDermott, 2020). Although spectral regularity indicates harmonic relations, it can be extended to simply regular intervals of spectral components that deviate from multiple integer harmonics of F0 but have equal spacing or that include shifting of phases on each component, while holding grouping effect and behavioral relevance (Roberts and Bailey, 1996; So et al., 2020).

Natural sounds, such as human speech, animal vocalizations, and many environmental sounds, are comprised of complex spectrotemporal modulations (Chi et al., 1999; Elliott and Theunissen, 2009; McDermott and Simoncelli, 2011). Modulation energy in speech, for example, is captured by a decaying low-pass distribution below ∼16 Hz temporal modulation frequencies and ∼2 cycle/octave spectral modulation frequencies (Chi et al., 1999; Elliott and Theunissen, 2009). Word recognition is severely impaired when temporal modulation frequencies below ∼8–12 Hz are unavailable (Drullman et al., 1994; Elliott and Theunissen, 2009). This upper limit closely corresponds to the syllabic rate in speech (Greenberg et al., 2003; Pellegrino et al., 2011). Identifying syllables is essential for distinguishing words. The analysis of Western music revealed that temporal modulation for music is similar to speech; however, the peak energy is shifted down from 5 Hz for speech to 3 Hz for music reflecting the typical tempo (beats or rhythms) of music (Ding et al., 2017). Both for speech and music, most of modulation energy is <32 Hz, although rapid temporal modulations >50 Hz are critical for the perception of aspects such as pitch, lexical meaning, formant and timbre patterns, and their detections in noisy environments (Rosen, 1992; Shamma and Lorenzi, 2013).

Sound information is transmitted from the cochlea, via the medulla, pons, midbrain, and thalamus to the cortex. In parallel to the ascending pathways, descending pathways project information back to each stage (Winer, 2006; Souffi et al., 2021). Feedback projection activity can alter neural excitability (Villa et al., 1991; He, 1997, 2003; Lohse et al., 2020) and tuning properties for frequency (Yan and Ehret, 2002; Luo et al., 2011), intensity (Yan and Ehret, 2002; Ma and Suga, 2007), sound onset (Luo et al., 2008), sound duration (Ma and Suga, 2007), or sound source location (Nakamoto et al., 2008) as has been shown for the cochlear nuclei, inferior colliculus (IC), or medial geniculate body (MGB). Behavioral experiments have demonstrated that descending feedback projections modulate perceptual abilities, including detection and discrimination of sound frequency (Guo et al., 2017), harmonicity (Homma et al., 2017), and location (Bajo et al., 2010) (for review; Lohse et al., 2019).

**FIGURE 2:**
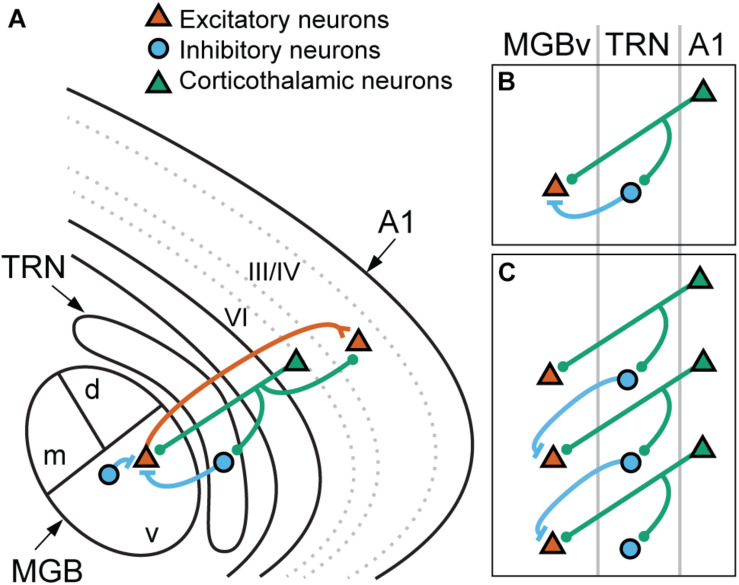
Corticothalamic connections to the lemniscal thalamus mediated via the thalamic reticular nucleus. **(A)** Schematic illustration of thalamocortical and corticothalamic connections. Thalamocortical neurons (red) in the ventral division of medial geniculate body (MGBv) project mainly to the neurons in layer III/IV of the primary auditory cortex (A1). The collaterals innervate to the thalamic reticular nucleus (TRN) but are not depicted in the diagram for simplicity. Corticothalamic neurons (green) in layer VI project back to the MGBv with the collaterals to the TRN and layer III/IV of A1. TRN and local GABAergic neurons provide inhibitory inputs (blue) to MGBv neurons. **(B,C)** Potential roles of the corticothalamic feedback. **(B)** Corticothalamic neurons modulate gain of MGBv neurons by regulating the balance of monosynaptic excitatory and disynaptic inhibitory inputs. **(C)** Corticothalamic neurons modulate tuning properties of MGBv neurons by lateral inhibition. The collaterals onto TRN spread along neighboring neurons with similar but not identical BFs, and the excitatory and inhibitory inputs to the MGBv neurons come from different corticothalamic cells. d, dorsal MGB; m, medial MGB; v, ventral MGB.

The projection from layer VI neurons of primary auditory cortex (A1) to the ventral division of medial geniculate body (MGBv) is one of the major feedback pathways (Figure 2A). A1-MGBv corticothalamic feedback projections have small excitatory terminals (Ojima, 1994), which are thought to act as “modulators” that regulate gain and firing patterns in the thalamus (Sherman and Guillery, 1998, 2011). The balance of excitation from the corticothalamic neurons and inhibition from thalamic reticular nucleus (TRN) neurons, which receives collateral projections from thalamocortical and corticothalamic neurons, modulates the tuning properties, gating and firing patterns of MGBv neurons (Zhang and Suga, 2000; Tang et al., 2012; Guo et al., 2017; Lohse et al., 2020).

In this review, we first introduce the anatomy and physiology of A1-MGBv corticothalamic feedback projections, then we summarize the feedback modulations of thalamic responses related to spectral and temporal information for speech and music processing. Finally, we explore perceptual effects of corticothalamic feedback, particularly on frequency and harmonicity analysis, and discuss how A1-MGBv corticothalamic feedback could contribute to our music and speech perception.

## A1-MGBv Corticothalamic Pathway

### Ascending Thalamocortical Projections

The main auditory nucleus of the thalamus, i.e., the MGB, is subdivided into three distinct areas. The ventral division is part of the lemniscal pathway and shows a tonotopic organization, in which characteristic frequencies of neurons are arranged in a dorsolateral to ventromedial topographic gradient from low to high frequencies (Aitkin and Webster, 1972; Calford, 1983; Figure 2A). MGBv mainly receives inputs from the ipsilateral central nucleus of the inferior colliculus (CNIC) (Calford and Aitkin, 1983; LeDoux et al., 1985; Rouiller and de Ribaupierre, 1985) with glutamatergic but also GABAergic connections (Winer et al., 1996; Peruzzi et al., 1997) and projects to A1 (Andersen et al., 1980a; Lee and Winer, 2008). The cerebral cortex consists of different types of cells that are functionally organized into a laminar structure, and MGBv neurons mainly target layer III/IV of A1 but also other cortical layers and especially layer I (Huang and Winer, 2000; Kimura et al., 2003; Smith et al., 2012; Vasquez-Lopez et al., 2017; Xie et al., 2018). MGBv also receives a small amount of input from the shell regions of IC (Kudo and Niimi, 1980; LeDoux et al., 1985) and innervates other “core” cortical areas that usually express a tonotopic organization and, to a lesser extent, “belt” areas that receive major non-lemniscal thalamic inputs (Andersen et al., 1980a; Morel and Imig, 1987; Redies et al., 1989; Velenovsky et al., 2003; De La Mothe et al., 2006; Polley et al., 2007; Lee and Winer, 2008; Hackett et al., 2011; Saldeitis et al., 2014). Thalamic inputs sourcing different locations of MGBv in the caudal-to-rostral dimension project to the targeted locations of the core cortical areas in the ventral-to-dorsal dimension without much overlap (Storace et al., 2010, 2011; Read et al., 2011), suggesting distinct functional segregations within the lemniscal thalamocortical system.

By contrast, the medial and dorsal divisions of the auditory thalamus (MGBm and MGBd) are considered non-lemniscal areas and do not have a clear tonotopic organization compared to MGBv (Calford, 1983; Rouiller et al., 1989; Hackett et al., 2011). Comparative studies of these three divisions and non-lemniscal corticothalamic feedback have been reviewed previously (Bartlett, 2013; Lee, 2015). Briefly, MGBm mainly receives inputs from the external cortex of the IC and projects to all layers of the auditory cortex whereas MGBd is innervated by dorsal cortex of IC and projects to layers I, III/IV, VI of belt and parabelt regions and only weakly to core auditory areas (Andersen et al., 1980b; Kudo and Niimi, 1980; LeDoux et al., 1985). Non-lemniscal MGB also receives inputs from superior colliculus (Holstege and Collewijn, 1982), and sends outputs to the striatum and amygdala (LeDoux et al., 1991), integrating multimodal sensory and emotional information (Weinberger, 2011).

### Descending Corticothalamic Projections

The tonotopic organization can also be a hallmark for descending pathways and is preserved in descending lemniscal axons. In principle, lemniscal cortical regions project back to subcortical lemniscal stations, whereas non-lemniscal cortical regions target non-lemniscal stations (Winer, 2006). Thus, lemniscal corticothalamic neurons project back from layer VI to MGBv with minor inputs to MGBm and MGBd (Andersen et al., 1980a; Rouiller and de Ribaupierre, 1985; Bajo et al., 1995; Budinger et al., 2013) in parallel to the tonotopic organization of lemniscal ascending thalamocortical projections (Redies et al., 1989; Rodrigues-Dagaeff et al., 1989; Velenovsky et al., 2003; Read et al., 2008; Hackett et al., 2011; Storace et al., 2011). The information flow is organized in a layer specific manner; layer VI corticothalamic neurons send collaterals to layer III/IV (Figure 2A; Ojima, 1994; Llano and Sherman, 2008), where the axon terminals of thalamocortical projections are mainly found. The A1-MGBv corticothalamic projections are mainly ipsilateral and form the focal topographic reciprocal connections; however, a minority showed non-reciprocal inputs (Winer and Larue, 1987; Winer et al., 2001). In addition, A1 also projects to MGBd as a descending feedforward projection, which is thought to transmit sound information from layer V of A1 to non-primary higher cortices (Ojima, 1994; Llano and Sherman, 2008). This cortico-thalamo-cortical connection is essential for corticocortical communication and processing higher-order sound features (Lee, 2015; Williamson and Polley, 2019).

The corticothalamic feedback projections from layer VI and the cortico-thalamo-cortical signaling from layer V are distinguished by their morphological characteristics. Tracer injection studies showed that the first-order corticothalamic feedback neurons in layer VI have small distal terminals with thin axons and convergent endings in MGBv while the higher-order corticothalamic feedforward neurons in layer V have large boutons in MGBd (Rouiller and Welker, 1991; Ojima, 1994; Bajo et al., 1995; Bartlett et al., 2000; Winer and Prieto, 2001). These two thalamic terminal types are characteristic for Class1 and Class2 glutamatergic projection neurons, respectively, based on structural and physiological properties (Sherman and Guillery, 1998, 2011). Corticothalamic feedforward neurons in layer V are classified as Class1, which have been characterized as “drivers” due to their function of relaying information to the cortex. On the other hand, corticothalamic feedback neurons in layer VI are classified as Class2 or “modulators” controlling how relay neurons transmit their information. They activate type I metabotropic glutamate receptors, which are identified as a characteristic of modulator synapses, and show paired-pulse facilitation with small excitatory post-synaptic potentials (EPSPs) in MGBv and layer IV cortical neurons (Bartlett and Smith, 2002; Lee and Sherman, 2009; Lee et al., 2012). A recent study also supports the idea that corticothalamic feedback projection neurons in layer VI are “modulators” with high selectivity of information flow while corticothalamic feedforward projections from layer V are “drivers” that integrate and transmit information (Williamson and Polley, 2019). Additionally, layer VI corticothalamic neurons can be activated by preparatory motor actions that trigger reward and auditory inputs in behaving mice, supporting the modulatory role for active listening (Clayton et al., 2021). Overall, layer VI corticothalamic neurons modulate MGBv neurons by regulating their excitability, voltage gated conductance, and synaptic potentials.

### Inhibitory Inputs and Intracortical Local Circuits

In addition to MGBv, corticothalamic feedback neurons send their axonal projection terminals to layer IV and the TRN (Jones, 2007; Sherman and Guillery, 2013; Figure 2A). TRN consists of GABAergic cells and is located between the cortex and the thalamus wrapping the thalamic nuclei with a sheet structure. The auditory sector is identified at the posterior-ventral part of TRN and receives thalamocortical collaterals (Rouiller et al., 1985; Villa, 1990; Conley et al., 1991). MGBv neurons receive inhibitory inputs from TRN in addition to local and IC GABAergic inputs (Winer and Larue, 1996; Winer et al., 1996; Arcelli et al., 1997; Peruzzi et al., 1997; Zhang et al., 2008; Clarke and Lee, 2018). The proportion of local GABAergic neurons in MGBv present species-specific variations, being almost absent in rodents and ∼25% of MGBv neurons in carnivores and primates (Winer and Larue, 1996). Corticothalamic projections to MGBv and TRN generally preserve topographic connections (Conley et al., 1991; Kimura et al., 2005; Cotillon-Williams et al., 2008); therefore, the feedback from layer VI shapes thalamic tuning by modulating the balance between converging excitation and inhibition.

In the cortex, the thalamocortical inputs are received mainly in layer III/IV. The thalamorecipient neurons then innervate the upper or supragranular cortical layers. Recent studies suggest that corticothalamic projection neurons in layer VI induce overall gain change across all cortical layers by recruiting local fast-spiking inhibitory neurons to modulate cortical oscillation (Olsen et al., 2012; Bortone et al., 2014; Kim et al., 2014; Guo et al., 2017). Taken together, this corticothalamic loop arborizing through A1, MGBv, and TRN shapes the receptive field structures and modulates information flow both in the thalamus and cortex and, thus, embodies a crucial aspect of the thalamocortical interface.

## Cortical Manipulation Onto MGBv Neurons

### Changes in Spectral Tuning

In laboratory experiments, single frequency tones (“pure tones”) are generated as a sound with a sinusoidal waveform. A simple spectral tuning curve is typically obtained by measuring the change in response to each pure tone at different combinations of frequency and intensity. Neurons in MGBv predominantly show narrow tuning curves, indicating high frequency selectivity, and short response latencies (cats: Calford, 1983; Morel et al., 1987; Miller et al., 2002; rats: Bordi and LeDoux, 1994; guinea pigs: Edeline et al., 1999; mice: Anderson and Linden, 2011; marmosets: Bartlett et al., 2011), Overall, MGBv neurons show sharper frequency tuning compared to A1 neurons by 0.1–0.3 octave (Miller et al., 2002; Bartlett et al., 2011). It is unknown at this time whether the thalamic frequency tuning properties in anesthetized or passively listening animals are predominantly inherited from the midbrain or shaped by local circuits with or without corticothalamic feedback.

**FIGURE 3:**
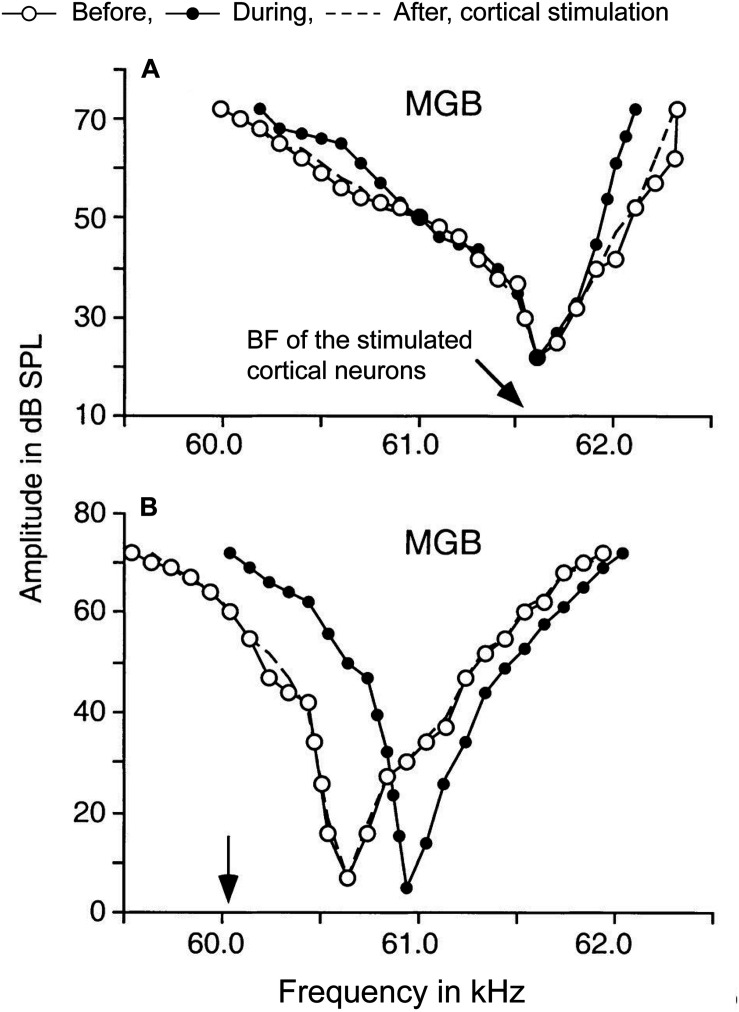
Corticothalamic modulation of frequency tuning curves in MGBv neurons. A focal electrical activation of cortical neurons in the Doppler-shifted constant frequency processing area of Jamaican mustached bats evoked changes of frequency tuning curves in MGBv neurons. The black arrows indicate the best frequencies (BFs) of cortical neurons stimulated. The tuning curves were estimated before (white circles), during (black circles), and after (dashed lines) the cortical stimulation. **(A)** A MGBv neuron showed sharpening of the tuning curve when cortical neurons that had matched BF to the recipient MGBv neuron. BF of the MGBv neuron did not change. **(B)** Another MGBv neuron showed a reduction of responses around BF of the MGBv neuron when BF of stimulated cortical neurons is unmatched to the recipient MGBv neuron. The tuning curve was shifted away from the BF of stimulated cortical neurons with increasing responses outside of BF of the MGBv neuron. The changes were transient only lasting 1–2 h after the 7-min cortical stimulation (Adapted and modified with permission from Zhang and Suga, 2000; Figure 5).

Thalamic tuning properties can be altered by manipulating the corticothalamic activities in layer VI. Modulation of frequency tuning by corticofugal activity is dependent on the relationship between the best frequencies (BFs), i.e., the frequency that evokes the highest firing rate, of subcortical and cortical neurons (e.g., Zhang and Suga, 2000; Figure 3). Observed tuning changes in MGBv neurons following A1 manipulation fall into two main categories. First, when the BF of a corticofugal neuron is matched to that of the recipient thalamic neuron, the frequency tuning at the latter is sharpened by facilitation of the responses at BF and reduction of responses to frequencies away from BF (Figure 3A). Conversely, when the BF of corticofugal neurons differs from that of the affected MGBv neuron, responses at the BF of MGBv neuron are reduced and responses to surrounding frequencies are enhanced, shifting the tuning curve away from the BF of the stimulated corticofugal neuron (Figure 3B). Cortical focal electric stimulation has been shown to induce both types of changes in MGBv of bats (Zhang and Suga, 2000; Tang et al., 2012). Similar changes are also induced in MGBv by electrical stimulation of the cholinergic nucleus basalis or by behavioral conditioning, and are abolished with inactivation of the auditory cortex (guinea pigs: Edeline and Weinberger, 1991; bats: Zhang et al., 1997; mice: Zhang and Yan, 2008; Luo et al., 2011; Nelson et al., 2015). This suggests that the effects on subcortical activity are controlled by the balance of local excitation and inhibition including the influence of various neuromodulators.

These two types of response changes potentially could arise from different, converging projections. When corticothalamic and corticoreticular pathways are strictly reciprocal, the balance of excitation and inhibition on a thalamic relay cell could be modulated by monosynaptic inputs from layer VI corticothalamic cells and disynaptic inputs from TRN mediated via the collateral of the same corticothalamic cells (Figure 2B). When the collaterals spread to the neighboring thalamic cells that have different tuning properties (Figure 2C), the corticoreticular pathway could sharpen thalamic receptive fields by lateral inhibition. Although the cascades of information flow from the periphery to the first-order thalamic nuclei differ among different sensory systems, the anatomy and physiology of thalamocortical and corticothalamic neurons are generally comparable (Rouiller and Welker, 2000; Sherman and Guillery, 2013). In the somatosensory system, most corticothalamic and corticoreticular projections are organized in a reciprocal manner and contribute to gain control on the thalamic cells, and some projections diverge to neighboring cells that have different tuning properties and show lateral inhibition (Temereanca and Simons, 2004; Li and Ebner, 2007; Lam and Sherman, 2010; Crandall et al., 2015). Orientation tuning of visual thalamic receptive fields also is shifted by focal pharmacological activation in layer VI of the primary visual cortex (Wang et al., 2016). Thus, a shift of tuning properties is a universal aspect of corticothalamic interactions and likely is mediated by disynaptic inhibition via diverged corticoreticular projections.

The ability to alter receptive field tuning may enable an attentive listener to focus on a specific speaker, other sound attributes of current interest, or adjust to changes in the sound environment by adapting to sound statistics in the environment similar to what has been demonstrated in cortical neurons (Fritz et al., 2003; Holdgraf et al., 2016; Homma et al., 2020). A major purpose of the modulation of receptive field selectivity by corticothalamic projections could be an increased discrimination ability, e.g., for frequency and musical pitch, to enhance scene analysis in complex sound environments.

### Gain Changes

In addition to the modulation of tuning properties, it was observed that lemniscal corticothalamic feedback generally facilitates excitability of MGBv neurons (cats: Ryugo and Weinberger, 1976; Villa et al., 1991; He, 1997; guinea pigs: He et al., 2002; Yu et al., 2004; mice: Guo et al., 2017; Lohse et al., 2020; marmosets: Zhang et al., 2021). Deactivation of the auditory cortex decreases the spontaneous firing rate in MGBv neurons (Ryugo and Weinberger, 1976; Villa et al., 1991; but see Zhang et al., 2021) while cortical activation enhances their responses to sounds (He, 1997; He et al., 2002; Guo et al., 2017). The effect is, however, heterogeneous, and a minority of MGBv neurons can show suppression. The proportion of excitation and inhibition differs among species due to different density of local GABAergic interneurons in MGBv (Winer and Larue, 1996; Arcelli et al., 1997). Since local inhibitory cells as well as long-range inhibitory inputs from the TRN play a key role in shaping thalamic processing, the interspecies differences in inhibitory capacity may bear on potential differences in the ability to modify processing via corticothalamic inputs.

**FIGURE 4:**
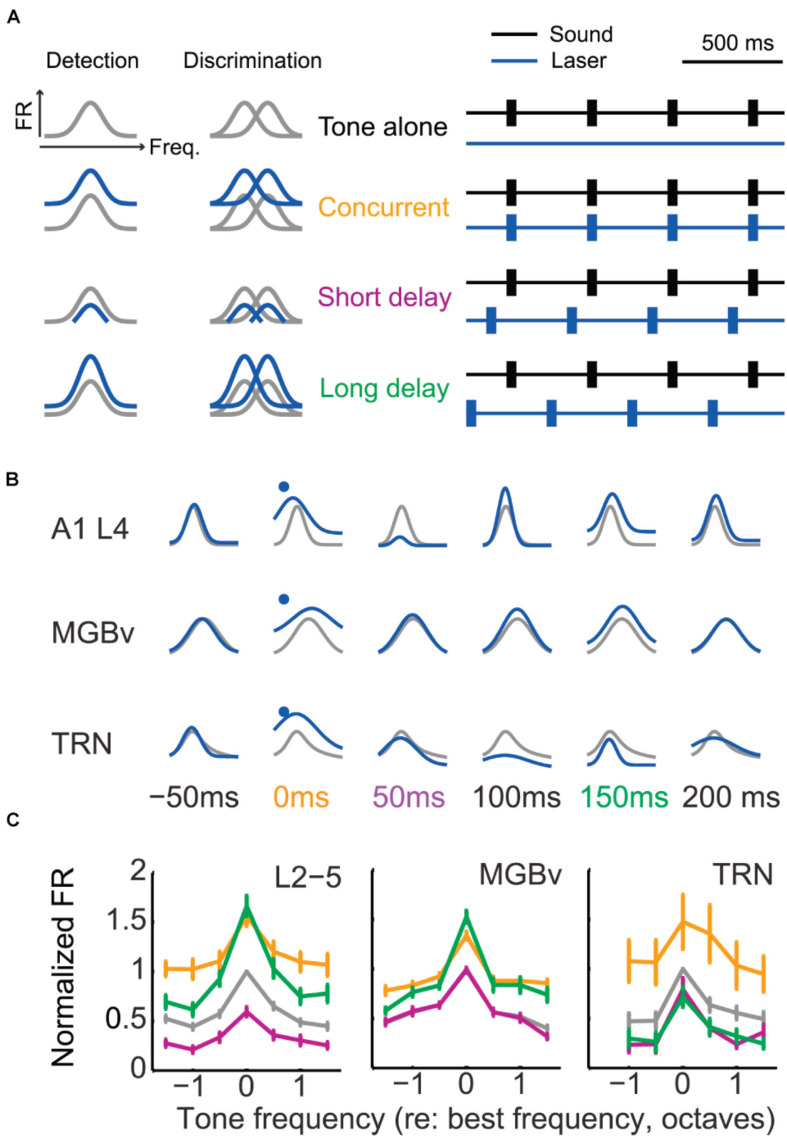
Corticothalamic gain control on frequency tuning curves of A1, MGBv, and TRN neurons. **(A)** Schematic of corticothalamic modulation on the turning curves of A1 cortical neurons and optogenetic laser stimulation paradigm. Lemniscal layer VI corticothalamic neurons were activated by expressing ChR2 in A1 bilaterally in Ntsr1-Cre mice and using pulses of blue laser light. Depending on the duration of the sound stimulation delay following laser activation of corticothalamic neurons in layer VI, the tuning curves were predicted to be modulated distinctively and to define tone detection and discrimination behaviors. The animals were trained to detect or discriminate sounds using an avoidance task. **(B)** Representative modulation effect on the tuning curves of A1, MGBv, and TRN neurons (gray, tone-alone; blue, tone-and-laser). Sound-evoked responses enhanced in A1 and MGBv neurons for the concurrent stimulation of tones and laser (orange). For the tone presentation with a short or long delay following the corticothalamic stimulation (purple or green), corticothalamic modulation effects differed in A1, MGBv, and TRN neurons, showing enhancement for one station while showing suppression for the others. **(C)** Tone-evoked firing rates (mean ± SEM) were normalized and compared to the values evoked for the BF of the tone-alone condition. When tone presentation and corticothalamic stimulation were concurrent, all A1, MGBv, and TRN neurons increased firing rates (paired *t* test, *p* < 0.05). When tones were presented with a short delay following laser stimulation, the firing rates decreased in A1 and TRN neurons (*p* < 0.05) but no change was found in MGBv neurons (*p* > 0.05). For the long delayed condition, A1 and MGBv neurons increased firing rates (*p* < 0.05), whereas TRN neurons reduced it (*p* < 0.05) (Adapted and modified with permission from Guo et al., 2017; Figures 4B, 5D,E).

A recent study showed that activation of corticothalamic neurons in layer VI enhanced or suppressed activity in A1, MGBv, and TRN neurons with a dependence on relative timing of optogenetic and sound stimulation (Guo et al., 2017; Figure 4). The authors further found that the facilitated activities in MGBv could improve performance on a behavioral frequency detection task by increasing sound-evoked responses whereas better frequency discrimination was more closely related to suppressed cortical stimulus response (Olsen et al., 2012; Bortone et al., 2014). These observations indicate that corticothalamic feedback modulations are diverse and dependent on stimulus and/or task context.

The gain control provide by corticothalamic feedback induces a change in tuning sharpness by shifting the overall excitability of MGBv neurons but generally without affecting BF. As anatomical and physiological studies have shown (see section “A1-MGBv Corticothalamic Pathway”; Guo et al., 2017), corticothalamic feedback modulates not only MGBv but also A1 and TRN. Furthermore, corticothalamic feedback via TRN inhibiting MGBv seems to determine whether A1 neurons respond to weak tones or not (Ibrahim et al., 2021). Future investigations will need to explore the joint effects of the combined thalamocortical-corticothalamic-corticoreticular-intracortical loop. In addition, forward and feedback effects must be studied in the context of natural stimuli, such as communication sounds, and with the consideration of specific task goals and motivations.

### Temporal Representation and Precision

MGBv neurons are highly sensitive to temporally fluctuating sound, such as amplitude modulated tones or noise. The majority of these neurons show strongly synchronized firing patterns to temporal modulations of 20–40 Hz, but phase-locking is typically limited to modulation frequencies below ∼60 to 200 Hz (guinea pigs: Creutzfeldt et al., 1980, cats: Rouiller et al., 1981; Miller et al., 2002, non-human primates: Preuss and Müller-Preuss, 1990; Bartlett and Wang, 2007, 2011). Those values are two to four times higher for MGBv neurons than for A1 neurons (Miller et al., 2002; Bartlett and Wang, 2007) reflecting the progressively reduced ability of envelope synchronization along the ascending pathway (Joris et al., 2004). While ∼40% of MGBv neurons with BFs mostly >1.5 kHz show exclusively a rate code, another ∼40% of MGBv neurons show both synchronized responses and increased firing in a single neuron dependent on repetition rates, and the proportion was five times larger than that for A1, suggesting that MGBv is a transition stage for this computation (Bartlett and Wang, 2007; but see Yin et al., 2011). For some thalamocortical transmissions, the temporal code is transformed to a rate code due to the synaptic interactions, in which excitatory and inhibitory inputs both inherit a temporal code from MGBv but the spiking response loses synchronization if they are in-phase (Bartlett and Wang, 2007; Gao and Wehr, 2015). Furthermore, higher auditory cortical fields encode temporal fluctuations predominantly in firing rate, with the exception of very low temporal modulation frequencies (Scott et al., 2011; Hullett et al., 2016; Johnson et al., 2020).

MGBv neurons respond strongly to species-specific and other animal vocalizations, and the response preferences are consistent with the values estimated by conventional artificial stimuli synthesized to represent acoustic properties contained in vocalizations (Creutzfeldt et al., 1980; Symmes et al., 1980; Philibert et al., 2005; Suta et al., 2007). Phase-locking to the F0 of harmonic vocalization is observed when neurons have the capacity of phase locking to pure tones in the range of the F0 (Wallace et al., 2007). The temporal spike pattern of MGBv neurons more closely matches the spectrogram of vocalizations and shows higher decoding performance than A1 neurons (Huetz et al., 2009; Souffi et al., 2020), suggesting that some information of the vocalization content is still present in a temporal code at the thalamic level.

Thalamocortical neurons have been shown to switch between two distinct firing modes: “burst” and “tonic” (Steriade et al., 1993; Sherman, 2001; Llinás and Steriade, 2006). When the principal cells are depolarized by incoming inputs and switch into “tonic mode,” voltage-gated T-type Ca^2+^ channels become inactivated. In this mode, the firing patterns show a linear relationship to the input strength and spikes occur with high temporal precision (Mease et al., 2014; Hasse and Briggs, 2017); therefore, the “tonic mode” is more suitable for sound discrimination sound. After a period of depolarization, cells switch into “burst mode” by re-activation of inward Ca^2+^ current. In this mode, cells are hyperpolarized and prone to produce spikes with lowered threshold and less temporal precision. Thus, the input-output relationship is highly non-linear (McCormick and Feeser, 1990; Zhan et al., 1999) and is thought to be more helpful for sound detection (Hu et al., 1994; Bartlett and Smith, 1999; but see Massaux et al., 2004).

Stimulation of corticothalamic neurons in layer VI induces depolarization in MGBv neurons shortly after hyperpolarization (Bartlett and Smith, 2002; Yu et al., 2004), indicating that corticothalamic feedback can induce MGBv neurons to act in the “tonic mode” as has been seen in other sensory systems (Mease et al., 2014; but see Denman and Contreras, 2015). Furthermore, corticothalamic activation reduces adaptation to rapid repetitive stimulation as excitatory and inhibitory synaptic depression differs between the first-order visual/somatosensory thalamus and TRN (Mease et al., 2014; Crandall et al., 2015). In the auditory thalamus, corticothalamic activity seems to reduce adaptation to less-salient modulated noise since inactivation of corticothalamic neurons blocked the reduction (Kommajosyula et al., 2021). Therefore, corticothalamic neurons may be helpful for maintaining precise temporal responses in sequential or rapidly fluctuating sounds, which is characteristic of music and speech, and especially in less-salient sounds.

The assumption that corticothalamic neurons modulate temporal precision in MGBv is mainly based on intracellular recording of responses to pure tones or broadband noise. The likelihood of tonic and burst modes in the thalamus is affected by the different brain states of waking, sleep, attentiveness, and anesthesia (Weyand et al., 2001; Massaux et al., 2004; Gent et al., 2018). In the auditory thalamocortical system, burst mode is suppressed when spectrotemporally modulated broadband noise is presented compared to spontaneous or tone-driven activity (Miller and Schreiner, 2000). Further investigations are needed that use stimulus with more naturalistic modulations and behaving animals in order to dissect how the corticothalamic feedback affects the perception of naturally modulated sound, such as speech and music.

Overall, corticothalamic neurons in layer VI have potentially three major physiological functions in their effects on MGBv neurons: (i) refining the receptive field structure, (ii) modulating response gain, and (iii) controlling temporal precision, by regulating the balance of monosynaptic excitation and disynaptic inhibition. These three aspects are not operating independently, and the causes and effects of these interactions remain to be explored in more detail. This is relevant in the context of the next question, namely how corticothalamic modulations shape sound analysis and auditory perception.

## Effects of Corticothalamic Feedback on Sound Perception

### Frequency Analysis

Although some physiological functions of lemniscal auditory corticothalamic neurons have been gradually revealed in the past decades, it remains to be determined how the feedback affects hearing abilities in common, natural tasks. It has been technically challenging to selectively target corticothalamic neurons for recording and/or manipulations while, at the same time, measuring perceptual attributes in awake, behaving animals. Although a recent study using optogenetic phototagging in awake mice showed that layer VI corticothalamic neurons have narrower frequency tuning and higher selectivity of information flow compared to layer V corticofugal neurons (Williamson and Polley, 2019), the studies for corticothalamic modulations discussed above were largely based on recordings under anesthesia. It has been postulated that corticothalamic feedback is, in particular, required for more complex sound processing in behaving animals since ablation of auditory cortex revealed performance deficits in discrimination of frequency modulated tones but not for simple frequency tones (Ohl et al., 1999; Ono et al., 2006). However, none of these studies could dissect the separate roles of cortico-cortical vs. thalamo-cortical vs. cortico-thalamic contributions. More recently, layer specific electrical microstimulation showed that modulation of signal detection and cortical frequency processing do appear to involve recurrent cortico–thalamo–cortical interactions (Happel et al., 2014; Saldeitis et al., 2021). As mentioned above (see section “Gain Changes”), optogenetic activation of corticothalamic neurons in layer VI modulated sound detection and frequency discrimination abilities of mice (Guo et al., 2017). It demonstrated that the physiological effects of layer VI corticothalamic feedback, such as refining tuning curves and controlling gain of MGBv and A1 neurons, can contribute to modulate frequency perception. Collectively, the present understanding is that layer VI corticothalamic neurons that project to MGBv neurons can affect spectral perception and are likely critical for perceptually demanding situations.

### Harmonic Structure Analysis

#### Mistuning Detection

We hypothesized that corticothalamic feedback from layer VI is particularly important for complex sound processing and focused our study on harmonic structure. Harmonicity perception is potentially regulated by all the three physiological effects of layer VI corticothalamic feedback proposed above. Harmonicity is a strong grouping cue in speech/music perception and scene analysis. Sounds with a harmonic structure are typically perceived as one single entity associated with a specific pitch (Roberts and Bregman, 1991; Roberts and Bailey, 1993), despite containing many different frequency components. When background noise has harmonic structure, detection of foreground sound is improved (Deroche and Culling, 2011; Steinmetzger and Rosen, 2015; Guest and Oxenham, 2019). In order to explore roles of harmonicity, inharmonic sound stimuli have been generated in several different ways by perturbing a regular frequency interval of harmonic structure. It can be achieved by simply shifting all the harmonics to lower or higher frequencies to the same degree (i.e., the frequency interval no longer matches to F0 but regular), stretching out frequency intervals of harmonic components (i.e., each interval differs in the harmonic series), or randomly shifting each harmonic in a small degree. Since the former two cases preserve some levels of spectral regularity, this moderately contributes to fused perception (Roberts and Brunstrom, 1998, 2001). Using harmonic and randomly inharmonic synthetic vowels, harmonicity is shown to improve the segregation of concurrent vowels (Culling and Darwin, 1994; de Cheveigné, 1995; de Cheveigné et al., 1997). When inharmonicity is artificially introduced by jittering each harmonic of speech, the accurate segregation of speech from competing speech or speech-like noise is impaired (Popham et al., 2018).

**FIGURE 5:**
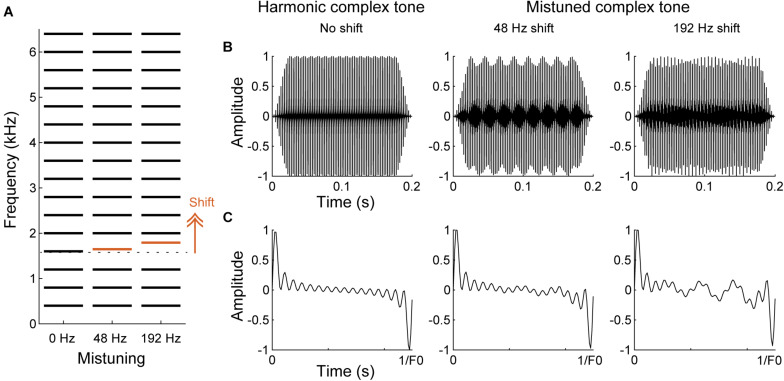
Spectral and temporal cues in harmonic and mistuned complex tones. **(A)** Schematic of spectral structures of harmonic and mistuned complex tones comprised a F0 of 400 Hz with 16 harmonics. 4th harmonic (red) is shifted to higher frequencies for the mistuned complex tones by 48 or 192 Hz. **(B,C)** The waveforms of the complex tones shown in A for the overall tone duration **(B)** and for one cycle of the period of the harmonic complex tone **(C)**. Due to the mistuned harmonic, additional temporal fluctuations emerge for the mistuned complex tones.

One broadly studied inharmonic tone paradigm is a mistuned complex tone, which comprises a harmonic shifted to lower or higher frequency in an otherwise harmonic complex tone (i.e., “mistuning”) (Figure 5). Specifically, the shifted component can be heard as standing out as a separate tone for low frequency harmonics or produce a sensation of roughness in the sound quality for high frequency harmonics (Moore et al., 1985, 1986; Hartmann et al., 1990). Perception of the shifted component as a separate tone is dependent on the frequencies of shifted components and degrades for higher F0s (Hartmann et al., 1990; Gockel and Carlyon, 2018). Based on the cochlear filtering model, a harmonic is assumed resolved when it falls in a single filter bank, while it is considered unresolved when several harmonics excite the same filter (Houtsma and Smurzynski, 1990; Shackleton and Carlyon, 1994). Thus, mistuning may be detected as a deviated spectral component in a spectral template of resolved harmonics (“harmonic template”) that is expected to be a series of multiple integers of the F0 of a harmonic sound (“spectral cue”) (Goldstein, 1973; Terhardt, 1974; Lin and Hartmann, 1998). Alternatively, the sensation of roughness or “beating,” which is produced by an interaction of adjacent frequency components within the same cochlea filter, has been thought to help detect disruption of harmonicity (Assmann and Summerfield, 1994; Culling and Darwin, 1994; but see de Cheveigné, 1999). Detecting an inharmonic sound may also be assisted by the temporal excitation patterns synchronizing to the envelope fluctuations (“temporal cue”) (Licklider, 1951; Meddis and Hewitt, 1991a, b). Both, spectral and temporal cues can be used for detecting a change of harmonicity.

In terms of harmonicity perception by animals, they have been found to be sensitive to harmonic structures (Kalluri et al., 2008) and can perform behavioral tasks for F0 judgment (Tomlinson and Schwarz, 1988; Walker et al., 2009; Osmanski et al., 2013) and mistuning detection (Lohr and Dooling, 1998; Klinge and Klump, 2009; Homma et al., 2016). Smaller animals are more likely to rely on temporal cues due to their generally broader cochlea filters (Sumner et al., 2018; Walker et al., 2019).

#### Neural Responses to Harmonic Complex Sounds

Neural responses can encode both spectral and temporal cues to harmonic and inharmonic complex sound (for review; Micheyl and Oxenham, 2010). At the auditory nerve, neurons respond to individual frequency components and can follow the F0 of complex tones with phase-locked responses up to ∼1 kHz (Horst et al., 1986; Cariani and Delgutte, 1996; Sinex et al., 2003; Cedolin and Delgutte, 2005). At higher stations of the central auditory system, temporal cues are degraded, and sound information is integrated. For harmonic sounds, neurons in IC and A1 increase firing rate when resolved harmonics are close to the neurons’ best frequencies and both stations show some phase-locking to the envelope periodicity (F0) for unresolved harmonics (Schwarz and Tomlinson, 1990; Steinschneider et al., 1998; Fishman et al., 2013; Su and Delgutte, 2019, 2020). The phase-locking limits to the F0, however, decrease tenfold from IC to A1. For inharmonic sounds, the neurons show phase-locking to the fine structure of the envelope as well as the periodicity of interactions between mistuned and neighboring harmonics (“beating”) (Sinex et al., 2002, 2005; Fishman and Steinschneider, 2010; Homma et al., 2017). While synchronized responses can be observed regardless of the distance between a mistuned harmonic and a frequency that MGBv or IC neurons are tuned to, changes of temporal patterns are weaker for A1 neurons when a mistuned harmonic is far away from a tuned frequency. In addition, the changes of firing rates occur to inharmonic sounds. The neurons increase their firing rates compared to the responses to the harmonic sound that has the same spectral components except the mistuned harmonic. Although a proportion of the neurons shows opposite decreasing trend, it results in enhanced responses for IC, MGBv and A1 neurons on average. Similar to temporal patterns, firing rates to mistuning are specifically enhanced in A1 when a mistuned harmonic is closer to a neuron’s tuned-frequency, and the frequency specific changes of temporal patterns and firing rates in A1 are thought to correlate with “standing-out” perception of a mistuned harmonic in humans (Fishman and Steinschneider, 2010). It is unknown whether thalamocortical and/or corticothalamic projections contribute to form the specificity to the mistuned harmonic frequency in A1.

The corticothalamic modulation likely enhances spectral analysis of harmonicity in MGBv by sharpening the spectral tuning, i.e., increasing the sensitivity to small frequency shifts of mistuning. Furthermore, the corticothalamic modulation may assist detection of precise temporal excitation patterns of mistuning by switching to a “tonic mode” and improving temporal representation. Moreover, enhanced encoding of harmonic components and periodicities may refine the analysis of harmonic complex tones via spectral and temporal cues, respectively. Consequently, corticothalamic feedback may improve pitch discrimination.

In human and non-human primate auditory cortex, a population of neurons has been found to be specialized for harmonicity processing. Those neurons are excited by the F0 of a harmonic complex sound even when the actual F0 is omitted (“missing fundamental”) or by periodic broadband noise stimulus that evoke pitch sensation, and were identified at the low frequency border of A1 and neighboring core region as well as at the adjacent belt regions (human: Patterson et al., 2002; Penagos et al., 2004; non-human primate: Bendor and Wang, 2005). For humans, non-primary auditory cortex is particularly critical for detecting pitch saliency and changes in pitch (Patterson et al., 2002; Penagos et al., 2004). Neurons responding to a subset of harmonics in harmonic complex tones (“harmonic template”), independent from their responses to pure tones, are scattered throughout the core regions of non-human primate (Feng and Wang, 2017). No equivalent type of response has been unequivocally identified at subcortical stations. It remains to be explored how the F0 representation emerges in the auditory system, whether the transformation is achieved by subcortical or cortical processing, and whether corticothalamic feedback is required.

#### Selective Elimination of Corticothalamic Neurons

**FIGURE 6:**
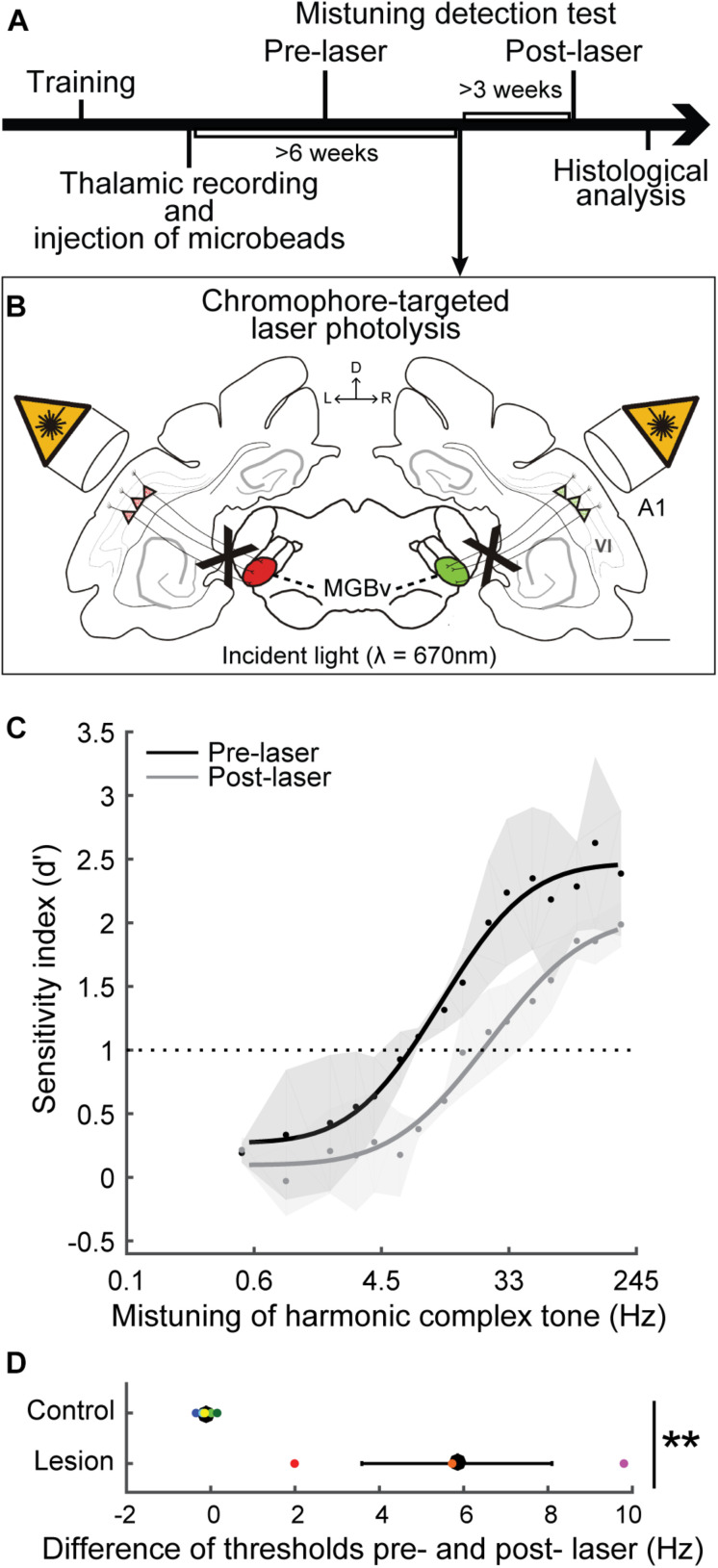
Selective elimination of corticothalamic neurons in layer VI impaired mistuning detection performance. **(A)** Ferrets were trained for a mistuning detection task using go/no-go behavior paradigm. The fluorescent microbeads conjugated with a light-sensitive chromophore were injected into bilateral MGBv (left, red; right, green) guided by thalamic recordings. **(B)** Apoptosis was induced for the retrogradely labeled corticothalamic neurons in layer VI by infrared laser light on A1. D, dorsal; L, left; R, right; VI, cortical layer 6. Scale bar = 1 mm. **(C)** The psychometric curves were plotted as a function of degree of mistuning (on a log scale). X-axis indicates less mistuning at the left and larger mistuning toward the right. Mistuning detection performance was impaired, shifting the curve toward larger mistuning after selective elimination of corticothalamic neurons (gray) compared to the baseline (black). **(D)** The difference of threshold before and after laser illumination was larger for the animals that received corticothalamic elimination compared to the control animals (two-tailed unpaired *t* test, **p < 0.01), supporting impaired behavior by a loss of A1-MGBv corticothalamic feedback. Individual animals are represented by colored dots (Adapted and modified from Homma et al., 2017; Figures 3D,F, 5B,C).

We have demonstrated that selective elimination of layer VI corticothalamic neurons using chromophore targeted laser photolysis impairs the ability of ferrets to detect mistuned complex tones (Homma et al., 2017). In the study, ferrets were trained in a go/no-go task to detect an inharmonic tone, which comprises the mistuned 4th harmonic in an otherwise harmonic complex tone of 16 harmonics with a F0 of 400 Hz (Figures 5, 6A). Then, fluorescent microbeads conjugated with a light-sensitive chromophore were injected bilaterally in MGBv, and >6 weeks later apoptosis was induced in the retrogradely labeled corticothalamic neurons in layer VI by focusing a infrared laser beam on A1 at the depth of the targeted layer (Figure 6B). About 60% of corticothalamic neurons were selectively eliminated. Mistuning sensitivity was measured behaviorally before and after the elimination of corticothalamic neurons, and the psychometric curve was constructed as a function of degree of mistuning (Figure 6C). Shifts of the 4th harmonic to a higher frequency ranged from 0 to 192 Hz. After the elimination of layer VI corticothalamic neurons, the psychometric functions were displaced to larger degrees of mistuning with reduced sensitivity (*d’*), indicating a deficit in discrimination ability. In addition, the threshold of detecting mistuning increased for the animals that received the corticothalamic lesion (Figure 6D). Although the lack of corticothalamic feedback could have reduced excitability of MGBv and decreased overall hearing sensitivity, there was no difference between lesion and control animals in the baseline performance of detecting an inharmonic tone with maximum degree of mistuning reinforced by a level difference of reference and target tones. These suggest A1-MGBv corticothalamic neurons are essential for successfully processing at least one important contributor to auditory scene analysis, namely for determining the harmonic structure of complex sounds. It remains to be elucidated how exactly the physiological changes of MGBv neurons by layer VI corticothalamic feedback contribute to mistuning detection related the temporal and spectral cues in mistuned complex tones. While reshaping of the receptive field structures is expected to improve spectral analysis of resolved harmonic components in MGBv, enhanced temporal precision in MGBv is predicted to refine temporal representation of periodicities in inharmonic complex tones. In addition, the modulation of gain titrates excitability of MGBv, which may control the focus to mistuned harmonic. All are plausible to increase the acuity of mistuning perception.

We postulate that corticothalamic feedback benefits other aspects of auditory scene analysis too. For example, enhanced signal detection by corticothalamic feedback seems to be robust for less salient inputs (Happel et al., 2014; Kommajosyula et al., 2021). Corticothalamic feedback may also contribute to signal-in-noise processing by amplifying weak foreground sounds by controlling the gain of MGBv neurons. Noise invariance also emerges from MGBv to A1 (Las et al., 2005; Rabinowitz et al., 2012; Schneider and Woolley, 2013; Souffi et al., 2020) and may be controlled by corticothalamic feedback. Finally, although speculative, the feedback may help in detecting onset synchrony, discriminating consonance and timbre, or assessing reverberation effects, by enhancing spectral and temporal processing in the MGBv.

## Speech and Music Processing in MGBv

In this section, we will discuss potential roles of lemniscal corticothalamic feedback in speech and music processing. The ability of modulating frequency and harmonicity perception suggests layer VI corticothalamic projections can be involved in regulating speech and music recognition. Although we will mainly focus on MGBv, we briefly summarize findings in the human auditory cortex for an overall view of the auditory forebrain system (for review; Zatorre and Schönwiesner, 2011; Leonard and Chang, 2014). Growing evidence indicates that the left hemisphere is specialized for speech processing while the right hemisphere is dedicated for music processing (e.g., Zatorre et al., 1994; Griffiths et al., 1999; Tervaniemi et al., 2000; Albouy et al., 2020). This asymmetry is supported by finer temporal representation for speech in the left and superior spectral representation for music in the right (Zatorre et al., 2002; Poeppel, 2003). Furthermore, cortical regions are hierarchically organized and functionally segregated. Spectrotemporal and phonological analyses for speech takes place in the left superior temporal gyrus (STG) and superior temporal sulcus (STS), which fall in the traditional “Wernicke’s area” (Scott, 2000; Davis and Johnsrude, 2003; Mesgarani et al., 2014). Human voice identities are encoded in the right STS (Belin et al., 2000; Schall et al., 2015). While low-level sound features are mainly represented in the core regions, high-order sound features in speech and music are encoded in non-primary regions (Norman-Haignere et al., 2015). Although frequency information is first processed in the lemniscal core regions with their tonotopic organizations, non-primary regions play key roles in pitch and melody perception (Patterson et al., 2002; Penagos et al., 2004; Albouy et al., 2020). Stronger and more selective responses to a single speaker in competing simultaneous speech are observed in non-primary areas than in A1 (Ding and Simon, 2012; Mesgarani and Chang, 2012; O’Sullivan et al., 2019). Thus, the auditory cortex, particularly high-order regions, are essential for phonological, semantic, and melodic processing of speech and music.

### Speech Processing

The human auditory thalamus is involved in some aspects of language processing. People with developmental dyslexia have difficulty in reading and writing and often exhibit impaired auditory and visual timing processing in various cortical and subcortical regions (for review; Ozernov-Palchik and Gaab, 2016; Stein, 2019). Some of those deficits could be traced to changes in the MGB (Galaburda et al., 1994; Diaz et al., 2012). Dyslexics often experience hindrances in auditory signal processing and sensorimotor processing. Psychoacoustic testing and auditory evoked potential studies show reduced sensitivities to discriminating temporally/spectrally modulated sound, or syllables (Stein and McAnally, 1995; Kraus et al., 1996; Menell et al., 1999; Goswami et al., 2002), and neuroimaging studies indicate deficits in rhythmic perception and audio-motor integration for dyslexics, which is in line with different neural phase alignment and consistency in the delta band compared to the control group (Hämäläinen et al., 2012; Colling et al., 2017). Functional magnetic resonance imaging (fMRI) revealed decreased activity in the left MGB for dyslexics in a syllabic discrimination task but not in passive listening condition (Diaz et al., 2012), supporting the possibility that top-down modulation, potentially corticothalamic feedback, differs between dyslexics and controls. Morphological examination showed that cells are smaller in the left than in the right MGB for dyslexics while no asymmetry is observed for control subjects (Galaburda et al., 1994). Dyslexics can exhibit malformation of cortical structures, “microgyria.” An animal model expressing microgyria also exhibited abnormal anatomical changes in the MGB as well as temporal processing deficits in behavioral tasks similar to human dyslexics (Fitch et al., 1994; Herman et al., 1997; Peiffer et al., 2002; Anderson and Linden, 2016). Irregular connections between MGBv and microgyri may perturb temporal processing in dyslexics. Although further investigations are required of the anatomical and functional changes in MGB that may contribute to phonological skills, the ability to process syllables or words with high temporal precision for spoken language in MGB appears to be closely linked to processing of written language.

Other evidence that the auditory thalamus is involved in speech processing arises from the modulation of thalamic activity by cognitive demands during speech-based tasks via top-down feedback (Alain et al., 2005; Christensen et al., 2008; von Kriegstein et al., 2008; Mihai et al., 2019). Although speech representation in the human auditory cortex has been extensively studied (for review; Zatorre and Schönwiesner, 2011; Leonard and Chang, 2014), investigations in the human thalamus with non-invasive methods have been challenging due to its relatively small volume and the deep anatomical position in the brain. Thus, the thalamic activities measured by positron emission tomography (PET) (Salvi et al., 2002) or fMRI (Alain et al., 2005; Tervaniemi et al., 2006; Christensen et al., 2008), often only reflect overall responses of the thalamic complex to speech signal. Although language processing is lateralized to the left hemisphere in the cortex (Zatorre et al., 2002), the presence of an equivalent thalamic lateralization is not fully established. Contrasting activations to a consonant-vowel-consonant-vowel pseudoword were observed for a change of duration in left thalamus and for a change of frequency in right thalamus (Tervaniemi et al., 2006). In addition, only the activity in right thalamus significantly differed between diotic and dichotic attentive listening conditions while both sides were activated for one to three syllable nouns compared to reversed speech (Christensen et al., 2008). Two other studies support left lateralization for sentence and vowel processing in thalamus (Salvi et al., 2002; Alain et al., 2005). The left thalamus was activated for trials with successful identifications of two vowels that were concurrently presented (Alain et al., 2005) further supporting that the thalamus is involved in F0 discrimination with top-down modulation. It is, thus, likely that some basic speech processing aspects are lateralized to the left thalamus.

**FIGURE 7:**
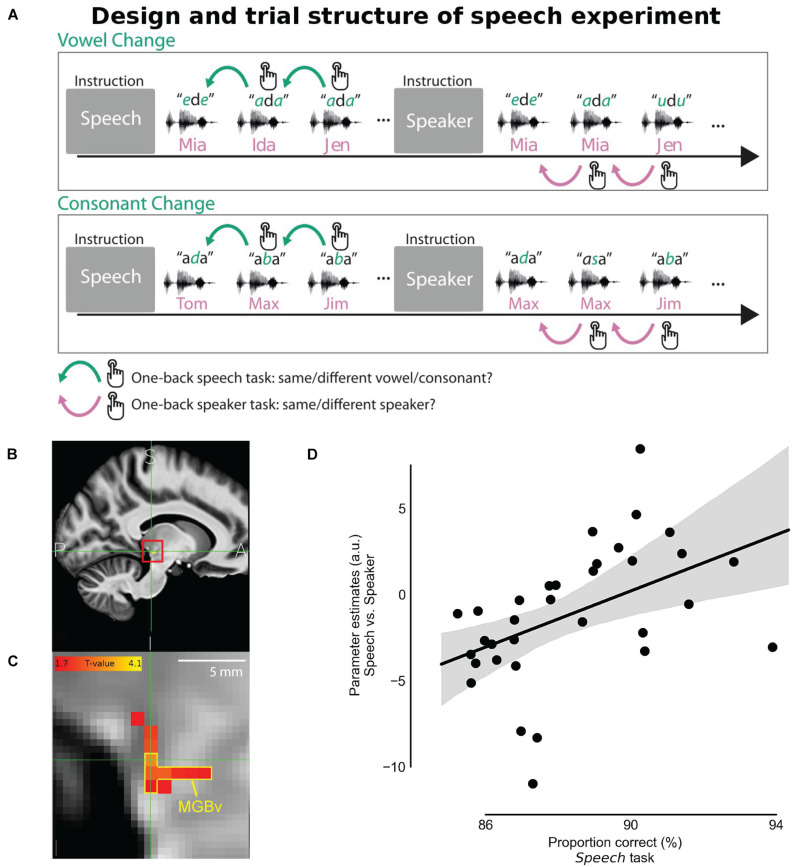
Task-dependent modulation in MGBv for speech recognition. **(A)** Behavioral task design. In the speech task, participants pressed a button when a syllable changed in the sequence of vowel-consonant-vowel-syllables stimulus. In the speaker task, using exactly the same stimulus, they were instead asked to report when speaker identity changed in regardless of syllable changes. **(B)** The panel shows the averaged structural image by fMRI (sagittal section) across participants on the human brain atlas. The location of the left MGB was estimated and indicated with the red square. A, anterior; I, inferior; P, posterior; S, superior. **(C)** Zoomed view of the red square in the panel B, denoting MGBv by the yellow contour. The strength of the correlation between the speech vs. speaker task contrast and the averaged behavioral correct performance rate in the speech task was depicted with hot color coding. **(D)** Speech vs. Speaker tasks activation at the left MGBv coordinates correlated with the proportion of correct responses in the speech task. The better behavioral performance in the speech task corresponded to the larger difference of BOLD response between speech and speaker tasks in the left MGBv. Dots represent individual participants. The line shows the best fit with the gray area indicating 97% bootstrapped confidence interval (Adapted and modified with permission from Mihai et al., 2019; Figures 1C, 6, 7).

Recent fMRI studies with finer spatial resolution successfully identified MGB and captured the tonotopic organization in its ventral division (Moerel et al., 2015; Mihai et al., 2019). The activated responses for discriminating speech signal was observed in both sides of MGB, however, activity correlated to the behavioral performance in a speech recognition task was only observed in the left MGB (von Kriegstein et al., 2008; Mihai et al., 2019). Mihai et al. (2019) assigned two different attentional tasks while listening to an identical set of sound stimuli. They asked participants to report a change in either the presented syllables or the speaker identity (Figure 7A). Changes between syllables were reported for the speech task while detecting change of F0s was used for the speaker task. Although there was no significant difference between speech and speaker task for the blood-oxygen-level-dependent (BOLD) responses in MGBv, the correlation between the evoked activity and the behavior performance of the speech task was only found in the left MGBv (Figures 7B–D). This suggests that top-down modulations enhance speech processing in the left MGBv. Then, introducing speech-shaped white noise as background, Mihai et al. (2021) showed that the enhanced top-down modulation on speech recognition is strongly observed in left MGBv when listening condition is challenging.

Corticothalamic feedback potentially strengthens spectral and temporal processing in MGBv and modulates frequency and harmonicity perception. Thus, syllable discrimination may have relied on enhanced frequency tuning and more precise spike representation via the feedback modulation to characterize individual syllables. In particular, attention could have modulated corticothalamic gain, affecting the excitability of MGBv neurons. Furthermore, although F0 discrimination did not show correlations with a lateralized change in activity in the study by Mihai et al. (2019), attentional modulation may be more relevant in harder discrimination tasks, such as segregation of competing simultaneous speech. We demonstrated that corticothalamic feedback was essential for processing spectral and/or temporal cues of the harmonic complex tones and detecting mistuning. A similar mechanism could assist speaker discrimination, mainly based on F0 discrimination using spectral structure and periodicity. F0 discrimination also helps to segregate foreground sounds from background “noise.” Thus, top-down modulation via corticothalamic feedback may assist segregating simultaneously presented signals, which is an essential function of auditory scene analysis.

It would be interesting to examine what features of speech are extracted at the lemniscal thalamus and how the processing is modulated by attention or task demands. The temporal resolution of magnetoencephalography (MEG), electroencephalography (EEG), or electrocorticography (ECoG) (<10 msec) is finer than fMRI (<5 s), and recent studies showed that activity in deep subcortical structures can be detected by MEG (Müller et al., 2019; Pizzo et al., 2019). Technical advances in temporal and spatial resolutions are expected to dissect in greater detail human speech processing mechanisms along the auditory pathways from subcortical to cortical stations. In the human auditory cortex, attentional switching between low and high frequencies changes the activated locations of fMRI voxels corresponding to the attended frequencies in the primary auditory areas (Da Costa et al., 2013). In animal studies using extracellular recordings, task engagement increases or decreases responses to the behavioral target sound and reshapes the receptive field structures in A1 neurons compared to passive listening and dependent on task difficulty (Fritz et al., 2003; Atiani et al., 2009; Lee and Middlebrooks, 2011; Niwa et al., 2012; Schwartz and David, 2018). Attentive modulation was, however, larger in the belt/parabelt areas than in the core areas (Atiani et al., 2014; Niwa et al., 2015; Elgueda et al., 2019). Thus, corticothalamic feedback may be a gate of top-down modulations for complex cortical processing, supporting precise acoustic representations in A1 via the aforementioned physiological functions. Future studies are necessary to elucidate how corticothalamic projections contribute to modulate representations of sound signals in A1 and higher cortical fields as well as in MGBv.

### Music Processing

Music processing studies in the human auditory system often involve a comparison between musicians and non-musicians since musical training is believed to induce plastic changes of structure and function in cortical and subcortical regions. Professional musicians tend to start receiving perceptual and motor training in their early childhood; therefore, structural and functional changes could reflect enhanced music processing. Plastic changes to musical training are indeed observed in the thalamus of musicians. Pianists have greater gray matter volume for the right thalamus (Vaquero et al., 2016), and drummers show increased oscillatory activities between the thalamus and premotor cortex/posterior parietal cortex (Krause et al., 2010). Furthermore, the thalamus is involved in musical imagery, i.e., an evoked sensation of music without external source. The right thalamus was activated for this music imagination phenomenon (Goycoolea et al., 2007) and melody recall (Zatorre et al., 1996). In addition, the ventral thalamus was activated during melody or sentence generation (Brown et al., 2006). Pleasant feelings associated with music listening have been shown to activate the thalamus, especially the mediodorsal thalamus, which regulates emotional processing (Blood and Zatorre, 2001; Klepzig et al., 2020). Contrasting to the left-hemispheric lateralization for speech processing, these studies support right-hemispheric lateralization for music processing.

It is, however, still not well investigated how MGBv processes musical signals and what are the main functional roles of corticothalamic feedback for music processing. For example, increased activation to urban noise, including music, were observed in the MGB of schizophrenic patients, supporting a role for the thalamus in sensory gating (Tregellas et al., 2009). Subcortical auditory structures, MGB and IC, with a strong corticofugal input, showed greater synchronization and responses to pieces of music compared to a scrambled version of the music or ripple noise, suggesting top-down modulation specific to music perception as opposed to basic sound perception (Abrams et al., 2013; Jiang et al., 2013). The thalamus was more highly activated to changes of musical chords or timbre (different musical instruments), when they deviated from an expected musical flow (Koelsch et al., 2002), again pointing a role of expectation or surprise in guiding corticothalamic feedback.

Musical chords of Western music consist of multiple harmonic complex tones (>2); in other words, combinations of pitches/harmonics. If two tones are separated by 1 octave, the frequency ratio is 2:1 (“unison,” e.g., A, 220 Hz and 440 Hz), preserving harmonicity and resulting in sounding pleasant (“consonant”). A combination of two tones with the ratio of 3:2 (“perfect 5th,” e.g., C and G) maintains the regularity of spectral components; therefore, it is consonant and usually evokes positive valence emotions. The ratio of 6:5 (“minor third,” e.g., A and C) has imperfect consonance and association to sadness (a typical difference between major and minor cords). At the other extreme, if the frequency ratio of two tones is 16:15 (“minor second,” e.g., C and C#), it sounds unpleasant (“dissonant”) and can evoke all variety of negative valence emotions. Musicians showed refined representation of musical chords in the auditory brainstem response compared to non-musicians, suggesting that top-down modulation by corticofugal projections could optimize subcortical activities to efficiently process music (Lee et al., 2009). The sensation of roughness or beating has been thought to contribute to dissonance perception, and the difference of consonance and dissonance is reflected in the phase-locking in A1 to the frequency interactions of spectral components (Plomp and Levelt, 1965; Fishman et al., 2001). It seems, however, spectral regularity, i.e., harmonicity or periodicity, plays a key role while the judgment of pleasantness is also dependent on Western musical experience and cultural environment (Tramo et al., 2001; Bidelman and Krishnan, 2009; McDermott et al., 2010, 2016; Bowling et al., 2018). A recent study suggests that harmonicity octave judgment of melodic lines, a sequence of tones, shares a similar mechanism to fused perception for octave harmonic structures (Demany et al., 2021). Given its apparent importance for perception of harmonic structure, corticothalamic feedback may help to discriminate musical chords and perceive musical melodies. Moreover, it may potentially help in the perception of rhythmic activity and its coordination between cortex and thalamus (Lee, 2013; Musacchia et al., 2014).

High-resolution human imaging and recording techniques are expected to shed light on the role of thalamocortical activity for a variety of music-based sound aspects in the near future.

Finally, speech and music processing are closely related and show overlaps of their functions. For example, musical training can improve language processing for children, adults or patients with language disorders (for review; Kraus and Slater, 2015; Coffey et al., 2017). Elementary school children (∼8 years old) who received musicianship classes, which included lessons of pitch and rhythm identification, and instrumental classes for 2 years showed better ability to correctly hear out speech from speech-shaped background noise than the controls that had only 1-year training (Slater et al., 2015). Musical training improves not only pitch encoding at subcortical and cortical levels for music and speech (Schön et al., 2004; Wong et al., 2007) but also speech-in-noise performance with enhanced temporal and spectral representation of speech (Parbery-Clark et al., 2011; Kraus et al., 2014; Swaminathan et al., 2015; Zendel et al., 2015). Since non-invasive techniques used in those studies did not explicitly identify which subcortical stations were involved, it remains to be examined what neural circuits are contributing for the improvement. As one of major corticofugal projections, layer VI A1-MGBv corticothalamic feedback could enhance spectral and temporal encoding of music and speech in the thalamus although other corticofugal connections from layer V to MGB and IC also may be critical for top-down modulations and plastic changes to musical training.

## Clinical Relevance

Along with musical training, corticothalamic feedback modulation may generally reinforce experience-dependent sound processing. Although speech processing ability degrades with aging, which is associated with impaired temporal coding and altered inhibitory signaling in the auditory system (Caspary et al., 2008; Gordon-Salant et al., 2011; Richardson et al., 2013; Presacco et al., 2016), temporal precision is actually improved in the MGB of aged-animals, which could indicate a compensation for the degraded hearing abilities via top-down modulation (Kommajosyula et al., 2019, 2021; Quraishe et al., 2020). Aged-musicians showed less degraded performance on the tasks that typically decline with aging (e.g., signal-in-noise, gap or mistuning detection) (Parbery-Clark et al., 2009; Zendel and Alain, 2012), which may reflect compensation via enhanced corticothalamic feedback. Thus, understanding corticofugal modulation may guide rehabilitation and training schemes for hearing impaired patients and therefore have clinical relevance.

## Concluding Remarks

It has been proposed that corticothalamic neurons in layer VI (i) refine the receptive field of MGBv neurons, (ii) control gain of sound information flow, and (iii) increase temporal precision, by regulating the balance of excitation and inhibition. Lemniscal corticothalamic feedback can modulate the perception of frequency and harmonic structures, which is a basis for complex sound processing utilizing spectral and temporal cues and assisting auditory scene analysis of segregating concurrent speech or extracting signal from background noise. Task-related modulation was observed in human MGBv for speech processing particularly in noisy listening conditions. Although music processing in MGBv largely remains to be explored, corticothalamic feedback is expected to improve pitch perception and support musical appreciation. The future investigations will need to examine what aspects of speech and music corticothalamic feedback can modulate, but also to build up our understanding of the corticothalamic circuits including corticoreticular and intracortical pathways. The cutting-edge techniques of dissecting neural microcircuits in behaving animals and neuroimaging with finer spectral temporal resolutions in humans are expected to advance it. Ultimately, the better understanding of descending modulation may help improve rehabilitation for hearing impaired patients and musical training.

## Author Contributions

NH and VB both conceptualized the review. NH wrote the initial draft and prepared the figures. VB supported and reviewed the manuscript. Both authors made a substantial, direct and intellectual contribution to the review, edited and revised the manuscript, and approved the submitted version for publication.

## Conflict of Interest

The authors declare that the research was conducted in the absence of any commercial or financial relationships that could be construed as a potential conflict of interest.

## Publisher’s Note

All claims expressed in this article are solely those of the authors and do not necessarily represent those of their affiliated organizations, or those of the publisher, the editors and the reviewers. Any product that may be evaluated in this article, or claim that may be made by its manufacturer, is not guaranteed or endorsed by the publisher.
